# Lymphopenia Is Associated with Gross Target Volumes and Fractions in Hepatocellular Carcinoma Patients Treated with External Beam Radiation Therapy and Also Indicates Worse Overall Survival

**DOI:** 10.1155/2019/9691067

**Published:** 2019-10-27

**Authors:** Hai-Ge Zhang, Ping Yang, Tao Jiang, Jian-Ying Zhang, Xue-Juan Jin, Yong Hu, Jing Sun, Shi-Suo Du, Zhao-Chong Zeng

**Affiliations:** ^1^Department of Radiation Oncology, Zhongshan Hospital, Fudan University, Shanghai 200032, China; ^2^Department of Radiation Oncology, Luoyang Central Hospital Affiliated to Zhengzhou University, Luoyang, Henan 471000, China; ^3^Department of Liver Surgery, Zhongshan Hospital, Fudan University, Shanghai 200032, China

## Abstract

**Purpose:**

To investigate whether lymphocyte nadir induced by radiation is associated with survival and explore its underlying risk factors in patients with hepatocellular carcinoma (HCC).

**Methods:**

Total lymphocyte counts were collected from 184 HCC patients treated by radiotherapy (RT) with complete follow-up. Associations between gross tumor volumes (GTVs) and radiation-associated parameters with lymphocyte nadir were evaluated by Pearson/Spearman correlation analysis and multiple linear regression. Kaplan–Meier analysis, log-rank test, as well as univariate and multivariate Cox regression were performed to assess the relationship between lymphocyte nadir and overall survival (OS).

**Results:**

GTVs and fractions were negatively related with lymphocyte nadir (*p* < 0.001 and *p*=0.001, respectively). Lymphocyte nadir and Barcelona Clinic Liver Cancer (BCLC) stage were independent prognostic factors predicting OS of HCC patients (all *p* < 0.001). Patients in the GTV ≤55.0 cc and fractions ≤16 groups were stratified by lymphocyte nadir, and the group with the higher lymphocyte counts (LCs) showed longer survival than the group with lower LCs (*p* < 0.001 and *p*=0.006, respectively). Patient distribution significantly differed among the RT fraction groups according to BCLC stage (*p* < 0.001). However, stratification of patients in the same BCLC stage by RT fractionation showed that the stereotactic body RT (SBRT) group achieved the best survival. Furthermore, there were significant differences in lymphocyte nadir among patients in the SBRT group.

**Conclusions:**

A lower lymphocyte nadir during RT was associated with worse survival among HCC patients. Smaller GTVs and fractions reduced the risk of lymphopenia.

## 1. Introduction

Radiotherapy (RT) is a local treatment modality for inoperable liver cancer. RT contributes to systemic immunity with a double-edged sword [1]. It has immunostimulatory effects via increased release of tumor-associated antigens, radiation-induced neoantigens, increased expression of heat shock proteins, release of high-mobility group box protein, and recruitment of effector cells to the tumor microenvironment. Conversely, RT also has immunosuppressive effects via upregulation of programmed death domain ligand-1 (PDL-1), cytotoxic T lymphocyte antigen-4 (CTLA-4), and depletion of circulating lymphocytes and lymphoid progenitor cells from primary and secondary lymphoid organs [2, 3]. Lymphocytes are the most radiosensitive cells in the erythroid, myeloid, and lymphoid lineages. The impact of RT on reducing circulating lymphocyte counts has been known for decades. However, the potential association of RT with tumor control and overall survival (OS) outcomes remained largely unexplored until recently. Recognition that the immune system plays a vital role in tumor surveillance and the advent of immunotherapy have renewed efforts to preserve a pool of functioning lymphocytes in systemic circulation after RT [4].

A previous study reported that minimum absolute lymphocyte count (min ALC), Barcelona Clinic Liver Cancer (BCLC) score, and serum alpha-fetoprotein (AFP) are independent prognostic factors for the survival of patients with hepatocellular carcinoma (HCC) treated with RT [5]. Another study on HCC showed that mean spleen dose and spleen V5 significantly predict min ALC. In addition, spleen V5 correlates with reduced min ALC in patients with HCC [6]. However, additional studies are needed to understand the effects of RT on survival of patients with HCC. Improvements in radiation technology have produced various regimens with decreased target volumes and fractions but higher biologically effective doses (BEDs) for patients with HCC. The impact of dose, fraction, and therapeutic duration on circulating lymphocytes remains unclear, especially for the liver, which is surrounded by vital abdominal blood vessels.

The ALC includes the total number of T cells, B cells, and natural killer cells. In the clinical setting, ALC is considered to reflect nutritional status and may be a surrogate marker for human immunity. HCC-related viral hepatitis and cirrhosis may lead to hypersplenism, which potentially results in decreased white blood cell (WBC) and total lymphocyte counts. Thus, investigating the effects of different RT fractionations on peripheral circulating lymphocytes is important for HCC treatment. The aim of this study was to investigate variables in patients with HCC, and explore their relationship with lymphocyte nadir and their effects on predicting survival.

## 2. Methods

### 2.1. Patient Selection

**Figure 1:**
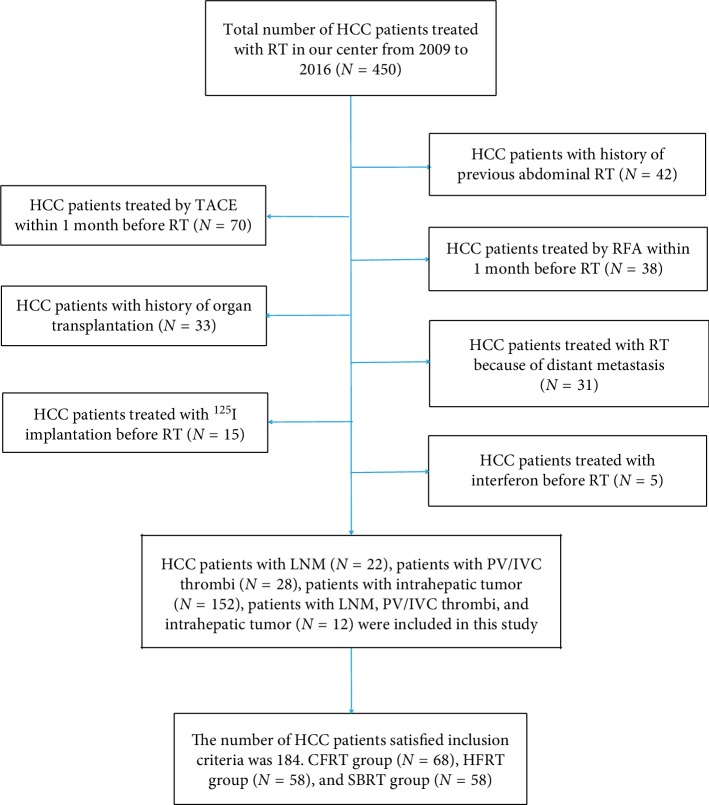
Flow chart.

This retrospective study was approved by the Ethics Committee of Fudan University Zhongshan Hospital (2011-235). Patients with HCC were consecutively entered into our study. We included 450 patients with HCC treated with RT who provided written informed consent at Zhongshan Hospital between August 2009 and December 2017. Patient diagnosis was confirmed by histology or clinical criteria [7]. In addition, patients met the following criteria: (1) HCC as primary cancer; (2) Karnofsky Performance Status (KPS) ≥ 80; (3) no previous abdominal radiotherapy; (4) no transcatheter arterial chemoembolization (TACE) or radiofrequency ablation (RFA) performed within 1 month before or after RT; (5) BCLC stage C only including portal vein tumor thrombi and abdominal lymph node metastases; (6) no history of organ transplantation; (7) WBC > 2.0 × 10^9^/L before RT; and (8) complete availability of follow-up data, medical records, baseline laboratory data, and at least two blood tests during RT. Patients were excluded if they had additional distant metastases or received interferon treatment. Finally, 184 patients with HCC were included in this study. The flow chart is shown in Figure 1.

All patients received pretreatment baseline evaluations, including physical examination, chest X-ray, contrast-enhanced abdominal computed tomography (CT) and/or magnetic resonance imaging (MRI), and positron emission tomography and computed tomography (PET-CT) scan if necessary. Peripheral blood draws from patients before and during RT, including complete blood cell count (CBC), liver function testing, serum chemistry, and serum AFP were performed to monitor the disease status.

### 2.2. Therapeutic Interventions

Patients were treated by conventional fractionation RT (CFRT), hypofractionation RT (HFRT), or SBRT according to doctor preference and patient economic status. CFRT was delivered at a total dose of 50.0–84.0 Gy at 1.8–2.5 Gy per fraction. HFRT was 58.5–91.1 Gy at >2.5 Gy to <6.0 Gy per fraction. SBRT was 79.0–119.0 Gy at 6.0–10.0 Gy per fraction. Total doses were translated into a biological effective dose using L-Q model with an HCC *α*/*β* = 10 (BED_10_) to allow comparison of doses by different fractionations. All patients were treated 5 days per week. Among 184 HCC patients, 137 positive for hepatitis B surface antigen (HBsAg) received antiviral therapy before and during RT. A 4D-CT simulator with contrast was used to evaluate liver motion with abdominal compression and to determine internal target volume (ITV). Gross tumor volume (GTV) was defined by an intrahepatic tumor, positive enlarged lymph node, and tumor thrombus. ITV was delineated as the sum of individual GTVs in the inspiration and expiration phases. The planning target volume (PTV) was defined as ITV with a margin of 5 mm in patients treated by three-dimensional conformal RT (3D-CRT) and intensity-modulated RT (IMRT) or 3 mm by image-guided IMRT (IG-IMRT).

### 2.3. Data Collection

Patient characteristics, including demographics and tumor status, were based on blood examinations and clinical data obtained prior to RT. The frequency of blood tests during RT ranged from 2 to 15 with a median of 4 times. ALC at pre-RT was defined as less than 2 weeks before the start of RT. Lymphocyte nadir was defined as the minimum value recorded during RT and within one month of the end of RT. Post-RT ALC was defined as ALC within 1 week after the end of RT. Post-pre ALC was defined as ALC at post-RT minus ALC at pre-ALC. ALC at the first and second follow-up was defined as ALC during 6–8 weeks and 12–16 weeks after finishing RT.

### 2.4. Radiation-Associated Parameters

The treatment plans were produced by physicists based on the dose parameters required by oncologists. Dose-volume parameters, including GTV, PTV, liver volume, fractionations, total dose, and the periods of RT, were collected from the planning system and treatment plans by physicists.

### 2.5. Follow-Up Evaluations

After RT, patients regularly returned for outpatient follow-up. The first visit occurred after 6 to 8 weeks, and then every 3 months during the first year, and every 6 months thereafter. Patients had blood draws to monitor CBC, liver function, serum chemistry, and AFP (if abnormally elevated before RT) and were also evaluated by physical examination. Abdominal MRIs and chest X-rays were generally added at the second follow-up. Chest CT, bone radionuclide imaging, and PET were performed if necessary. OS was calculated from the date of RT to the date of death or last visit (October 1, 2018).

Thirty-two (7.11%) patients who were lost to follow-up were censored at the last day they were known to be alive, and patients who remained alive were censored at the time of data cutoff. Missing data were handled in accordance with *Statistical Analysis with Missing Data* (the second edition was published in 2002) [8]. All assumptions for missing completely at random were met, and the analysis was carried out by data imputation [9].

### 2.6. Statistical Analyses

In this study, continuous variables are shown as medians, and ranges were compared with Mann–Whitney *U* test. Category variables are shown as frequencies and were compared with Pearson *χ*^2^ test. Receiver-operating characteristic (ROC) curve analysis was performed to determine the cutoff value for lymphocyte nadir and to calculate the optimal sensitivity and specificity for survival. Linear regression analysis and Pearson/Spearman correlation coefficients (*R*) were used to evaluate univariate associations between lymphocyte nadir and patient characteristics. Stepwise multivariate linear regression was performed to assess relationships of variables with *p* values less than 0.1 and lymphocyte nadir. Kaplan–Meier method and log-rank tests were applied to analyze and compare the OS rate. Univariate and multivariate Cox regression modeling were used to explore independent prognostic factors for OS. Stratified analyses were performed to compare OS between high and low LC groups based on related factors. Further stratified analyses were performed based on the BCLC stage to compare OS and the mean value of lymphocyte nadir. All statistical analyses in this study were performed using SPSS, version 24.0 (SPSS Inc., Chicago, IL, USA).

## 3. Results

### 3.1. Patient Characteristics

Between August 2009 and December 2017, 184 patients diagnosed with HCC who received RT at Zhongshan Hospital satisfied the criteria mentioned in Section 2.1 and were included in this study. Baseline demographics, tumor status, and characteristics of treatment are summarized in Table 1. There were 32 (17.4%) HCC patients with tumor thrombus and 22 (12.0%) with lymph node metastases (LNM) categorized as BCLC C stage. The median follow-up was 21.9 months (range, 1.3–107.3 months). The median age was 58 years (range, 22–87 years). Among all patients, 53 were treated with CFRT, 58 with HFRT, and 73 with SBRT. All patients finished RT without severe toxicities or complications.

### 3.2. Lymphocyte Counts during Radiation

**Figure 2:**
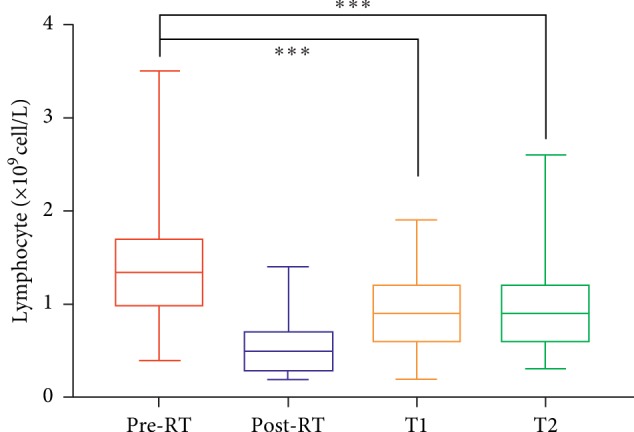
Alteration of lymphocyte count during radiotherapy (RT). Comparison of lymphocyte counts at pre-RT, post-RT, first follow-up (T1), and second follow-up (T2) (all *p* < 0.001).

As reported, lymphocyte counts generally declined during RT. The average ALC of pre-RT vs. post-RT was 1.33 vs. 0.50 × 10^9^ cells/L (*p* < 0.001) (Figure 2). During the period between the first and second follow-up evaluations, lymphocyte counts increased partially. However, there were significant differences between the pre-RT and the first or second follow-up visits (*p* < 0.001). Lymphocyte counts showed no significant difference between the first and second follow-ups (*p*=0.686) (Figure 2).

### 3.3. GTV and Fractions Are Associated with Lymphocyte Nadir

**Figure 3:**
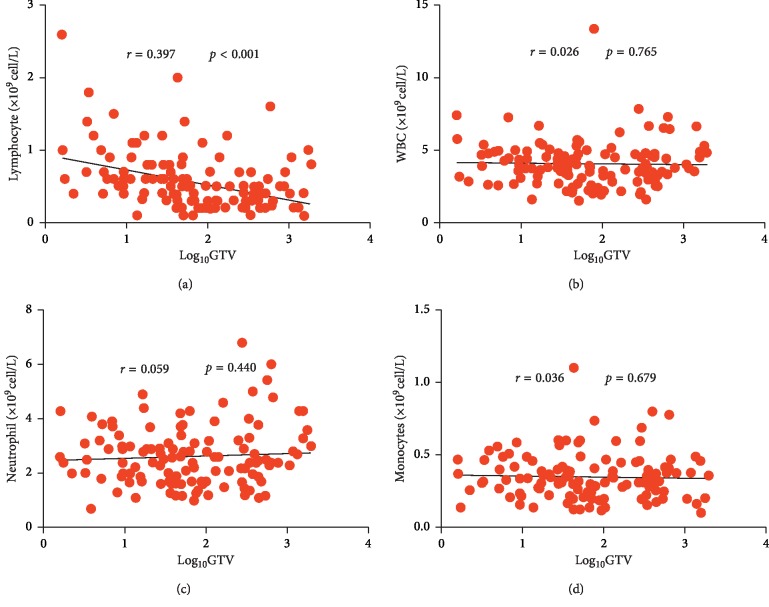
Correlations among lymphocyte (a), WBC (b), neutrophil (c), and monocyte nadir (d) (×10^9^ cell/L) during RT with log_10_GTV in patients with HCC.

We noted a significantly negative correlation between log_10_(GTV) and lymphocyte nadir (*r*=0.397, *p* < 0.001). Furthermore, we investigated total WBC (*r*=0.026, *p*=0.765), monocyte (*r*=0.036, *p*=0.679), and neutrophil counts (*r*=0.059, *p*=0.440). However, there was no significant correlation between log_10_(GTV) and the nadir of any other immune cell type (Figure 3). In order to evaluate whether this correlation was induced by radiation or existed before RT, we analyzed the pre-RT lymphocyte count. Generally, all patients had at least one blood draw before RT, and the most recent was analyzed. Compared with lymphocyte nadir during RT, pre-RT lymphocyte did not show significant correlation with log_10_(PTV) (*r*=0.057, *p*=0.521).

Table 2 lists variables for several demographic and pre-RT treatments significantly associated with lymphocyte nadir by univariate analysis. Specifically, age (*r*=0.179, *p*=0.025), RT fractions (*r*=0.296, *p* < 0.001), and BED (*r*=0.261, *p*=0.01) positively correlated with lymphocyte nadir. In contrast, Child-Pugh score (*r*=0.171, *p*=0.037), BCLC stage (*r*=0.198, *p*=0.014), fraction number (*r*=0.362, *p* < 0.001), tumor thrombus (*r*=0.178, *p*=0.026), LNM (*r*=0.160, *p*=0.045), and tumor size (*r*=0.195, *p*=0.015) negatively correlated with lymphocyte nadir. Finally, GTV (*p* < 0.001) and fraction number (*p*=0.001) were significantly associated with lymphocyte nadir in multivariate analysis.

### 3.4. Lymphocyte Nadir Is Associated with Survival

There were 68 (37.0%) patients alive at the last follow-up. The median survival of the whole cohort from the start of RT was 24.1 months, and the 1- and 2-year OS rates were 75.3% and 50.9%, respectively. Patients were classified into subgroups according to different variables (Table 3). Median survival was calculated by Kaplan–Meier analysis and log-rank test and is listed with the results of univariate and multivariate Cox analyses in Table 3. The median OS was worse in the group with low lymphocyte nadir compared with that of the group with high lymphocyte nadir. One- and two-year OS of the two groups were 56.7% vs. 80.3% and 28.4% vs. 55.7%, respectively (*p* < 0.001). There was no correlation between survival and gender, age, KPS, hepatitis B virus (HBV), TACE, RFA, pre-RT ALC, or post-pre ALC. Patients without tumor thrombus showed better OS (*p* < 0.001), as did those without LNM (*p*=0.018), with smaller tumor size (*p* < 0.001), treated with higher single-dose RT fractionation (*p* < 0.001), with higher BED (*p* < 0.001), with a lower fraction number (*p* < 0.001), with higher ALC post-RT (*p* < 0.001), with higher lymphocyte counts (*p* < 0.001), and with lower AFP (*p*=0.006).

**Figure 4:**
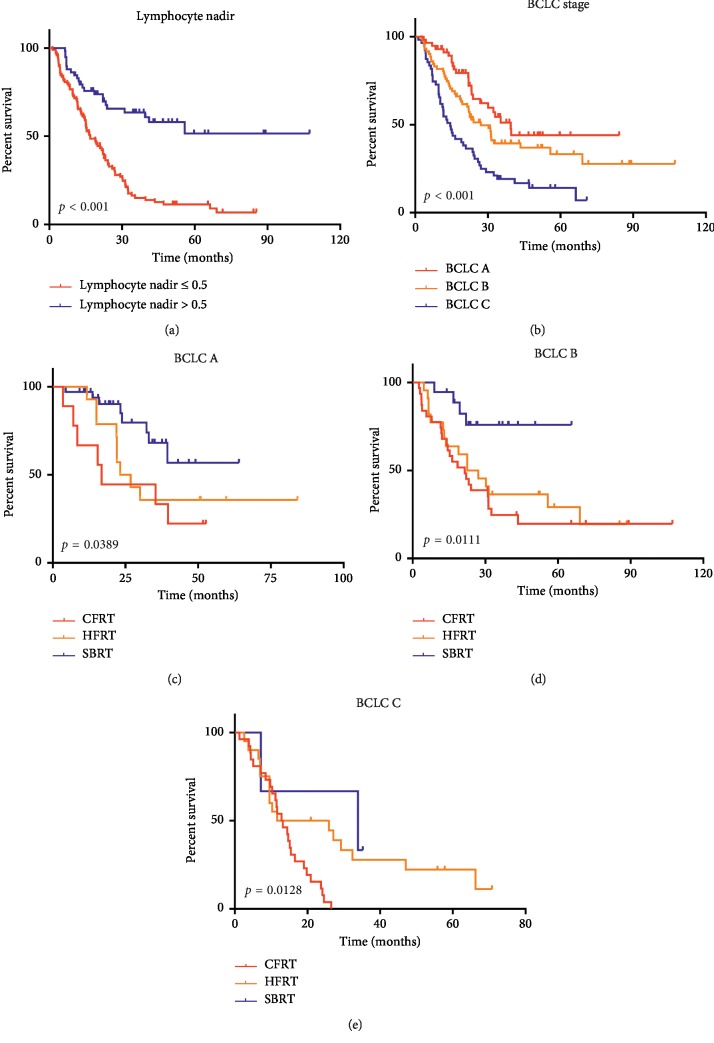
Survival curves according to independent prognostic factors of patients with HCC treated with RT (a). Patient survival analysis based on lymphocyte nadir, *p* < 0.001 (b). Patient survival analysis based on BCLC stage, *p* < 0.001, and stratified analyses based on BCLC stage A *p*=0.0389 (c). BCLC stage B, *p*=0.0111 (d). BCLC stage C, *p*=0.0128 (e).

**Figure 5:**
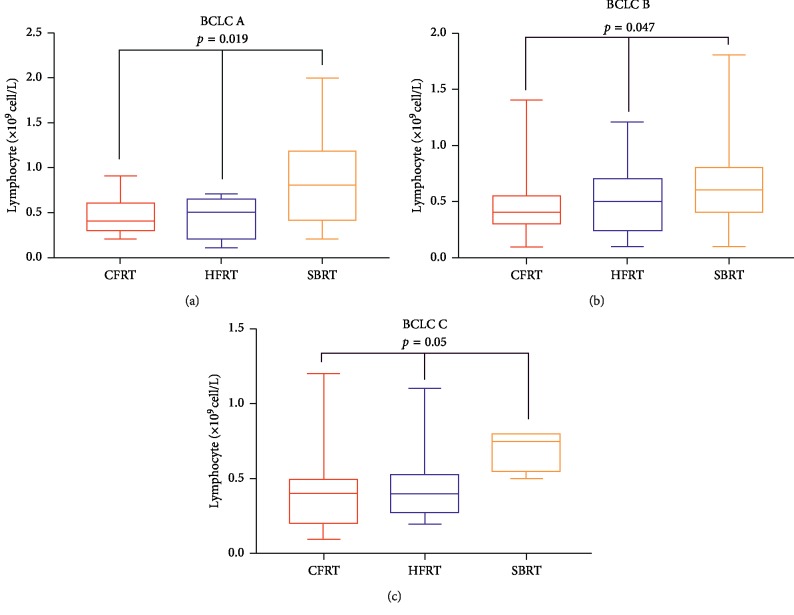
Comparison of lymphocyte nadir among CFRT, HFRT, and SBRT in patients with BCLC stage A, *p*=0.019 (a); BCLC stage B, *p*=0.047 (b); and BCLC stage C, *p*=0.05 (c), respectively.

We assessed the independent prognostic factors of patients with HCC among the aforementioned variables by Cox regression (Table 3). Variables with *p* < 0.1 were included in the multivariate analysis except the correlation factors of lymphocyte nadir (fraction number and GTV). Lymphocyte nadir was an independent prognostic factor for OS (Hazard ratio (HR) = 0.35; 95% confidence interval (CI) 0.19–0.63). When the significantly different variables were analyzed by multivariate analysis, post-ALC lost the ability to predict OS in HCC patients. Together with lymphocyte nadir, BCLC stage was an independent risk factor for predicting OS (Figure 4(a) and 4(b)). To analyze patient survival at the same level, we stratified by BCLC A, B, and C and compared the survival of the three groups. Patients treated by SBRT showed better survival in stratified analyses of BCLC A (Figure 4(c)) with *p*=0.0389, BCLC B (Figure 4(d)) with *p*=0.0111, and BCLC C (Figure 4(e)) with *p*=0.0128. LCs of patients in the SBRT group were significantly higher than those of the other two groups when stratified by BCLC stage A (*p*=0.019), BCLC stage B (*p*=0.047), and BCLC stage C (*p*=0.05) (Figure 5). We summarize patient characteristics among the three groups in Table 4.

### 3.5. Stratified Analysis Based on Fraction Number and GTV

The optimal threshold lymphocyte nadir to predict OS was confirmed by ROC curve analysis [10] and was 0.55 × 10^9^ cells/L. The specificity and sensitivity were 0.785 and 0.714, respectively. The area under the curve of lymphocyte nadir was 0.765. Patients were divided into a low lymphocyte nadir group (*n* = 99) and a high lymphocyte nadir group (*n* = 58).

**Figure 6:**
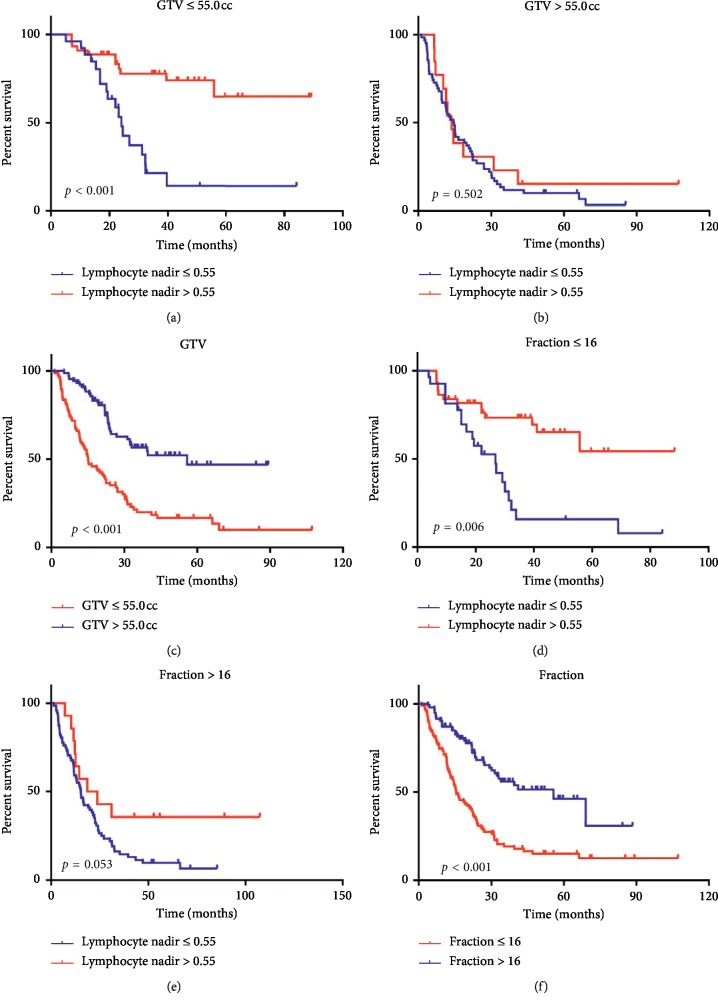
Survival curves according to independent prognostic factors. (a) Survival curves of patients in high and low lymphocyte nadir groups stratified by GTV ≤55.0 cc. The 1-year survival rate was 90.9% vs.77.7%; the 2-year survival rate was 88.5% vs. 53.0%; *p* < 0.001. (b) Stratified by GTV >55.0 cc (14.6 vs. 13.7; *p*=0.502). (c) Survival curve according to GTV groups (55.8 vs. 15.0 months; *p* < 0.001). (d) Survival curves of patients in high and low lymphocyte nadir groups stratified by fraction number ≤16. 1-year survival rate was 84.1% vs. 81.5%; 2-year survival rate was 73.4% vs. 52.3%; *p*=0.001. (e) Stratified by fraction number >16 (15.3 vs. 21.1 months; *p*=0.053). (f) Survival curve according to fraction number (55.8 vs. 15.4 months; *p* < 0.001).

Taking into account correlation factors of lymphocyte nadir, we excluded fraction number and GTV for multivariate analysis. In fact, they significantly affected OS. The median OS of the groups with fraction ≤16 group vs. fraction >16 group were 55.8 vs. 15.4 months (*p* < 0.001), respectively. The GTV >55.0 cc group showed worse OS than the GTV ≤55.0 cc group (55.8 vs. 15.0 months, *p* < 0.001). Therefore, we further examined the prognostic significance of lymphocyte nadir based on stratification of GTV and fraction number (Figure 6).

## 4. Discussion

Immunity and inflammation are crucial for the development and progression of liver cancers, immune surveillance, and treatment responses [11]. Lymphocyte count is a surrogate marker for the immunological status of patients and a prognostic factor for survival and recurrence for several cancers [12]. The impact of irradiation dose, fraction, and therapeutic duration on lymphopenia accompany multimodal cancer therapy. As the most radiosensitive cells of the hematopoietic system, lymphocytes residing within or circulating through a radiation portal are frequently depleted by radiation therapy. Radiation-induced reduction of circulating lymphocyte counts and the eventual lymphocyte infiltration of tumors impact OS outcomes and have revived interest in understanding the causes of treatment-associated lymphopenia to develop strategies to predict, prevent, and ameliorate this well-documented phenomenon [13, 14]. Although the mechanism was believed to be related to the irradiation of circulating blood, it remains unclear. Lymphopenia also appears after irradiation of the breast and brain, which contain little lymphatic tissue and bone marrow [15, 16]. Lymphocytes, the fundamental effector cells of the immune system, are sensitive to RT and recognize and kill tumor cells or release cytokines to activate the host immune system [17].

The causes of radiation-induced lymphopenia vary across disease sites. Larger radiation portals trigger greater depletion of circulating lymphocytes due to increased exposure of lymphocytes to radiation. The incidence of lymphopenia among patients with thoracic malignancies treated with radiation was in the range of 40–50%, comparable to that for head and neck cancer patients. Larger PTV, twice-daily fractionation, and higher radiation dose were associated with a higher incidence of lymphopenia. Grade 3-4 lymphopenia for abdominal tumors in the SBRT group compared with the conventionally fractionated concurrent chemoradiation group was 13.8% vs. 71.9% (*p*=0.001) at 1 month and 13.6% vs. 45.5% at 2 months (*p*=0.007) in locally advanced pancreatic cancer [18]. Severe lymphopenia at 2 months was found to be significantly associated with OS outcomes by multivariate analysis. The largest pools of resident lymphocytes that receive multiple-field irradiation during primary tumor treatment are the spleen and lymph nodes for abdominal cancer patients. The spleen, a secondary lymphoid organ and a reservoir of T and B lymphocytes, has a fenestrated endothelial lining with a slow circulating time for lymphocytes, most of which pass through the spleen. The liver, although neither a primary nor secondary lymphoid organ, harbors a large pool of circulating lymphocytes and may be a critical structure that is unintentionally irradiated during RT. Furthermore, unintentional RT to sites of lymphopoiesis, such as the spleen, portal hepatic lymph nodes, and gut-associated lymphoid tissue, which are secondary lymphoid organs, may also contribute to lymphopenia. Accordingly, we demonstrated that GTV and fractions were negatively related to lymphocyte nadir consistent with a report that GTV has the most essential influence on lymphocyte nadir in non-small cell lung cancer [19]. Nevertheless, association of GTVs with other immune cells, such as WBC, monocyte, and neutrophil nadir showed no correlation. Further, there was no association between pre-RT lymphocyte and GTVs. A larger target volume usually requires a richer blood supply, which increases the circulating lymphocytes included in the irradiation field. Bigger portal fields are required to cover larger GTVs, which causes irradiation beams to penetrate more normal abdominal tissue, including lymph nodes.

A model was created by Yovino et al. [20] to calculate the radiation dose received by circulating blood during external beam RT. It indicates that decreasing the target volume and the number of fraction can reduce the dose to circulating blood. In this study, we compared lymphopenia in groups treated with CFRT, HFRT, or SBRT. We found that lymphocyte nadir during RT was significantly different among the three groups (*p* < 0.001). SBRT, as a new regimen for HCC, seems to be able to protect lymphocytes. This result is consistent with a study in unresectable pancreatic cancer reported by Wild et al. [19]. However, in this study, BCLC stage as an independent prognostic factor is significantly different among the groups classified by CFRT, HFRT, and SBRT (*p* < 0.001). Therefore, it is necessary to discriminate potential influences on the association of OS and lymphocyte nadir. Stratified analyses based on BCLC stage showed the same results that OS in the SBRT group is the best of the three with significant differences. Furthermore, lymphocyte nadir of the SBRT group is also the highest one of the three. Several mechanisms may be involved in the effects of SBRT on immunity. Hypofractionated RT induces changes in the microenvironment. For example, higher doses, such as hypofractionated RT or SBRT, can promote the secretion of damage-associated molecules and may alter the tumor microenvironment to induce antitumor immunity [21–24]. SBRT can also promote the recruitment of immune cells to tumors. These immune cells play immune-modulating roles. For example, dendritic cells can mediate initiation of T cells and immune tolerance in the tumor microenvironment. SBRT may promote the effects of recruitment, maturation, and presentation of antigens to cytotoxic T lymphocytes (CTLs) [25–27]. After CFRT, lymphocytes are depleted because of high sensitivity to radiation [28].

In this study, multivariate analyses showed that lymphocyte nadir, but not pre- or post-RT lymphocytes, was a predictor of OS. In addition, BCLC stage was an independent prognostic factor in patients with HCC. In our study, patients in the BED >72 Gy group showed better median OS than those in the BED ≤72 Gy group (31.4 vs. 15.1 months, *p* < 0.001). This is in accordance with Dawson's report that patients with intrahepatic malignancies who received >70 Gy had better survival than those who received <70 Gy (16.4 vs. 11.6 months, *p*=0.0003) [29]. Finally, our investigation indicated that decreasing target volume and reducing fraction number spared the peripheral blood lymphocytes from RT-induced lymphopenia.

This is one of the first studies that reported the association of lymphopenia with RT for HCC [5]. Furthermore, we firstly investigated the difference of lymphopenia among CFRT, HFRT, and SBRT. There were several limitations in this study. It was a retrospective single-center study with a small number of patients. The frequency of blood draws and medical monitoring varied based on each patient's situation. We did not investigate the effects of other treatments after RT. Treatment strategy was determined according to doctor preferences and patient economic status. However, in fact, the economy is the primary factor. So far, SBRT has not been included in medical insurance in China. In addition, we didn't collect data on incidental irradiation of main lymphoid organs during RT in this study, such as bone marrow and spleen. Further prospective studies are needed to validate these findings.

## 5. Conclusion

Lower lymphocyte nadir during RT is associated with worse survival of patients with HCC. Smaller GTV and fraction number reduces the risk of lymphopenia.

## Figures and Tables

**Table 1:** Clinical characteristics of patients with HCC in the overall study cohort.

Characteristic	*N* = 184	*N* (%) or median (range)
Gender	Female vs. male	34 (18.5%) vs. 150 (81.5%)
Age (y)		58 (22–87)
KPS	≥90 vs. <90	168 (91.3%) vs. 16 (8.7%)
Child-Pugh	5 vs. 6 vs. ≥7	76 (41.3%) vs. 70 (38.0%) vs.28 (15.3%)
	Unknown	10 (5.4%)
BCLC stage	A vs. B vs. C	57 (31%) vs. 71 (38.6%) vs. 50 (27.2%)
	Unknown	6 (3.3%)
HBV	Negative vs. positive	47 (25.5%) vs. 137 (74.5%)
AFP (UI/ml)		28.1 (0–60500)
Tumor thrombus	Yes vs. no	32 (17.4%) vs. 152 (82.6%)
LNM	Yes vs. no	22 (12.0%) vs. 162 (88.0%)
TACE	Yes vs. no	109 (59.2%) vs. 75 (40.8%)
RFA	Yes vs. no	18 (9.8%) vs. 166 (90.2%)
Tumor size (cm)		4.1 (0.7–24.0)
ALC pre-RT (×10^9^/L)		1.3 (0.2–3.5)
ALC post-RT (×10^9^/L)		0.5 (0.1–2.2)
Lymphocyte nadir (×10^9^/L)		0.5 (0.1–2.6)
WBC nadir (×10^9^/L)		3.7 (1.5–13.4)
Mono nadir (×10^9^/L)		0.3 (0.1–1.1)
Neut nadir (×10^9^/L)		2.5 (0.7–12.2)
RT fractionation	CFRT vs. HFRT vs. SBRT	53 (37.0%) vs. 58 (31.5%) vs. 73 (31.5%)
BED (Gy)		75.0 (50.0–119.0)
Fractions		16 (5–35)
GTV (cc)		55.0 (1.6–1880.1)

KPS = Karnofsky Performance Status; BCLC stage = Barcelona Clinic Liver Cancer stage; HBV = hepatitis B virus; AFP = alpha-fetoprotein; LNM = lymph node metastasis; TACE = transcatheter arterial chemoembolization; RFA = radiofrequency ablation; ALC = absolute lymphocyte count; ALC pre-RT = ALC within two weeks before the start of RT; ALC post-RT = ALC within 1 week after the end of RT; ALC post-pre = ALC at post-RT minus ALC at pre-RT; WBC = white blood cell; Mono = monocyte; Neut = neutrophil; RT = radiotherapy; BED = biologically effective dose; and GTV = gross target volume.

**Table 2:** Univariate and multivariate linear regression associating baseline variables with lymphocyte nadirs during radiation treatment.

Variable	Regression coefficient	95% CI	*p* value
*Univariate analysis*			
Gender	−0.136	−0.294 to 0.022	0.090
Age	0.006	0.001 to 0.011	0.025
KPS	0.003	−0.011 to 0.017	0.681
Child-Pugh	−0.073	−0.141 to −0.004	0.037
BCLC stage	−0.1	−0.179 to −0.02	0.014
Tumor thrombus (yes/no)	−0.169	−0.318 to −0.021	0.026
LNM (yes/no)	−0.186	−0.368 to −0.004	0.045
TACE (yes/no)	−0.015	−0.138 to 0.107	0.805
RFA (yes/no)	0.027	−0.177 to 0.231	0.795
Tumor size (cm)	−0.019	−0.035 to −0.004	0.015
BED	0.007	0.003 to 0.012	0.010
Fractions	−0.016	−0.023 to −0.009	<0.001
Log_10_GTV	−0.205	−0.238 to −0.127	<0.001

*Multivariate analysis*			
Fractions	−0.029	−0.046 to −0.012	0.001
Log_10_GTV	−0.195	−0.291 to −0.099	<0.001

CI = confidence interval.

**Table 3:** Univariate and multivariate Cox regression analysis of survival among patient characteristics.

Variable	Univariate analysis		Multivariate analysis	
	HR 95% CI	*p* value	HR 95% CI	*p* value
Gender male vs. female	0.76 (0.47–1.25)	0.285		
Age (year) ≤58 vs. >58	0.82 (0.57–1.19)	0.298		

*KPS*		0.321		
80 vs. 90	0.93 (0.52–1.66)	0.802		
80 vs. 100	1.67 (0.67–4.21)	0.273		

*Child-Pugh score*		0.001		
5 vs. 6	1.84 (1.19–2.83)	0.006		
5 vs. ≥7	2.52 (1.49–4.26)	0.001		

*BCLC stage*		<0.001		<0.001
A vs. B	1.51 (0.92–2.48)	0.106	1.26 (0.68–2.31)	0.460
A vs. C	3.30 (2.00–5.44)	<0.001	3.68 (1.87–7.22)	<0.001

HBV negative vs. positive	0.99 (0.64–1.51)	0.950		
AFP (IU/ml) ≤28.1 vs. >28.1	1.79 (1.17–2.73)	0.007		
Tumor thrombus (no vs. yes)	2.69 (1.76–4.11)	<0.001		
LNM (no vs. yes)	1.82 (1.10–3.01)	0.020		
TACE (no vs. yes)	1.13 (0.78–1.65)	0.514		
RFA (no vs. yes)	0.55 (0.26–1.18)	0.125		
Tumor size (cm) ≤5.0 vs. >5.0	2.44 (1.68–3.53)	<0.001		
BED (Gy) ≤75 vs. >75	0.47 (0.32–0.69)	<0.001		
Fractions ≤16 vs. >16	2.79 (1.89–4.12)	<0.001		NI
ALC pre-RT (×10^9^/L) ≤1.3 vs. >1.3	0.81 (0.54–1.22)	0.313		
ALC post-RT (×10^9^/L) ≤0.5 vs. >0.5	0.41 (0.26–0.65)	<0.001		
Lymphocyte nadir (×10^9^/L) ≤0.5 vs. >0.5	0.31 (0.19–0.49)	<0.001	0.35 (0.19–0.63)	<0.001
GTV (cc) ≤55.0 vs. >55.0	3.00 (2.00–4.51)	<0.001		NI

NI = not included.

**Table 4:** Patient characteristics of conventional fractionationated RT (CFRT), hypofractionated RT (HFRT), and stereotactic body RT (SBRT) group.

Characteristic	CFRT (*n* = 68)	HFRT (*n* = 58)	SBRT (*n* = 58)	*p* value
*Gender*				0.976
Male	56 (82.4)	47 (81.0)	47 (81.0)	
Female	12 (17.6)	11 (19.0)	11 (19.0)	

Age (y), median (range)	57 (32–78)	58 (32–87)	60 (22–86)	0.301
*BCLC stage*				<0.001
A	9 (13.2)	14 (24.1)	34 (58.6)	
B	31 (45.6)	22 (37.9)	18 (31.0)	
C	27 (39.7)	20 (34.5)	3 (5.2)	
MV	1 (1.5)	2 (3.4)	3 (5.2)	

*HBV*				0.493
Negative	15 (22.1)	18 (31.0)	14 (24.1)	
Positive	53 (77.9)	40 (69.9)	44 (75.9)	

*Tumor size (cm)*				<0.001
≤5	23 (33.8)	23 (39.7)	47 (81.0)	
>5	45 (66.2)	34 (58.6)	8 (13.8)	
MV	0	1 (1.7)	3 (5.2)	

Pre-ALC (×10^9^/L), median (range)	1.3 (0.2–3.5)	1.3 (0.4–2.8)	1.4 (0.3–3.5)	0.886
Post-ALC (×10^9^/L), median (range)	0.4 (0.1–1.4)	0.4 (0.1–1.4)	0.6 (0.2–2.2)	<0.001
Lymphocyte nadir (×10^9^/L), median (range)	0.4 (0.1–1.6)	0.5 (0.1–1.2)	0.7 (0.1–2.6)	<0.001
BED (Gy), median (range)	64.8 (50.0–84.0)	75.0 (58.5–91.1)	86.4 (79.0–119.0)	<0.001
Fraction number	25 (16–35)	15 (10–25)	6 (5–10)	<0.001
GTV (cc), median (range)	190.5 (8.0–1711.3)	94.0 (2.2–1880.1)	15.2 (1.6–126.6)	<0.001

MV = missing value.

## Data Availability

The data used to support the findings of this study are included within the article.
